# Glasgow Medico-Chirurgical Society

**Published:** 1855-01

**Authors:** 


*Glasgow Medico-Chirurgical Society.*—Dr. Wilson, the President, introduced a discussion on Uterine Hæmorrhage, by detailing a case of Placenta Prævia, to which he had been called a few days ago, in consultation, and in which the vagina had been plugged before his arrival, with the view of arresting the flooding, which was considerable. He removed the plug, and found that the uterus had become gradually distended with blood. He introduced his hand, and soon completed the delivery by turning. The patient was doing well.* He gave it as his opinion, that, in most cases of this kind, it is better to allow the blood to flow from the vagina, than to use a plug, because the exact state of the case can then be known, while it is not unusual for the blood to continue flowing when the vagina is stuffed, and in these circumstances an

*Dr. Wilson has since learned that this patient died of phlebitis about three weeks after delivery.

immense mass of blood may collect in the vagina and uterus. This he had seen in several cases. When the quantity of blood becomes alarming, or even before this, we should, without waiting till the os tincæ becomes very dilatable, proceed at once to effect delivery by gradually causing dilatation by the introduction of the hand. Some of these cases may be lost by delaying too long, in expectation of the natural efforts being sufficient to effect dilatation; but when we have reason to suspect that a great deal of blood may be lost before the *os* has sufficiently relaxed, we should not hesitate at once to have recourse to artificial delivery.

Dr. J. G. Fleming lately met with a case of placental presentation, in which the vagina was firmly plugged, the hæmorrhage being considerable, and the os uteri firm, and very slightly dilated. An immense quantity of blood certainly collected in the uterus. Still he was not sure if it was right to condemn altogether the use of the plug; at an early stage of labor, when the detachment of the placenta is small, and the os uteri firm and little dilated, he thought that the introduction of a plug was useful; there would not be so much danger of further flooding, in consequence of the pressure exerted on the detached piece of placenta.

Dr. MacEwan had seen cases of the kind. He alluded to some instances of relaxation of the uterus after delivery, in which the organ lay loose and flaccid, and even the introduction of the hand failed to produce contraction. These cases should be carefully watched; but, strange to say, hæmorrhage was not so usual as might, under the circumstances, be anticipated.

Dr. Bell mentioned a cause of flooding after delivery, which he had noticed not unfrequently, and which he described as a patulous and relaxed condition of the *cervix* and lower part of the uterus. This, he believed, might often be found even when the fundus had become quite contracted. He considered it necessary, before leaving any case of labor, to make a vaginal examination, in order to ascertain the state of these parts, as the feeling communicated to the hand placed on the abdomen might be fallacious, and would, in cases of this sort, give no indication of the risk of hæmorrhage. This generally appears at first as a mere trickling from the vagina, in which, however, clots are soon formed, and so plug it up as to prevent an external flow of blood, which, however, accumulates internally, distending the uterus, before the general symptoms of flooding make their appearance. He considered that many cases of internal uterine hæmorrhage originated in this somewhat insidious manner.

Dr. R. G. Maxwell said he had met with three cases of presenting placenta, in which it was necessary to turn the child. The os uteri was so far relaxed in all of them before he made the attempt, that he was enabled, with moderate persistence, to pass his hand safely beyond it, and turn with ease. All the women had good recoveries; the children were dead—two of them had been so several days. Two of the cases were premature—one at 6½, the other at 8 months. In such cases the patient requires to be watched, and care taken that she does not lose so much blood as to endanger life before an attempt be made to turn. We may suspect internal hæmorrhage after delivery if the woman begins to yawn, and shows other symptoms of approaching syncope. On placing the hand on the lower part of the belly, if we find a large soft tumor, we should immediately insert our hand into the uterus, remove all the clots of blood, and endeavor to produce contraction at the fundus.

Dr. James Watson agreed with Dr. Fleming, that there were some cases in which we might employ a plug, especially at the commencement of labor, and until the *os* had somewhat yielded; but that, whenever symptoms called for it, turning should at once be performed. As hæmorrhage from placenta prævia often occurred long before it was possible to dilate the os uteri with safety, the plug was, in such cases, indispensable; but where the hæmorrhage took place in circumstances admitting of such interference, immediate delivery was, in his opinion, as a general rule, the proper practice. The last case of the kind he had, profuse hæmorrhage occurred two months before the patient was actually delivered; he used the plug, but after some days discontinued it. The patient was kept in bed all that time, without being permitted almost to move a hand or foot, and was most carefully watched. She did not lose much more blood till the time of her actual confinement. Then it did return, and he immediately delivered her. The plug was not used after the first attack. She recovered without a fault.

Mr. Lyon said, observation had taught him that the error of practitioners was the postponement of interference; trusting to hope in place of acting, until exhaustion became dangerous, and interference only hastened death. Whenever manual delivery was at all likely to be required, the earliest possible interference, consistent with prudence, was the safest rule. He knew, from a recent conversation on the subject in the old Medical Society, he was supported on this point by the deservedly high authority of Dr. James Wilson. To the various species of hæmorrhage mentioned by the various speakers, he added that from laceration inside of the fourchette. He had seen an instance of this kind where the bleeding continued for hours, seriously exhausting the patient; and the true source of which he was led to discover from the blood *trickling*, not *gushing*; when ligature of the open mouth of the artery in a trivial lesion inside of the fourchette soon righted matters.

Dr. Ritchie, being in the chair, (Dr. Wilson having been obliged to leave the meeting some time before,) summed up as follows:—The remarks of the different speakers appear to me to have embraced all the ordinary varieties of puerperal uterine hæmorrhage which are known, and one which I do not remember to have heard of before—that, namely, mentioned by Mr. Lyon, where a considerable bleeding was maintained, after the delivery of the child, from a lacerated artery in the situation of the fourchette. With bleeding from the rupture of a varicose vein in the labium I am acquainted, but not with that from an artery. Of the forms of uterine hæmorrhage arising from a separation of the placenta, before the birth of the child, reference has been made to cases in which the insertion of the placenta over the orifice was nearly complete, and to those in which it was partial only; and of bleeding after delivery, examples have been spoken of, in which it occurred from irregular contraction of a portion of the body of the uterus on the retained placenta, or, after the expulsion of the after-birth, on a clot, or on some still attached membranes; and also of hæmorrhage taking place, with a firmly-contracted fundus uteri, from relaxation of the orifice and neck of the organ.

In regard to the first form, or that where the bleeding appears in the latter months of pregnancy, from the necessary expansion of the orifice of the womb, the great practical difficulty is to determine the proper time to deliver. When the bleeding commences weeks before the natural period of parturition, is frequently repeated in moderate quantity, and your patient resides at a distance from you, and is not in labor, it is really not easy to decide. I am sure of this, however, that, should you resolve to delay, it is an unsafe practice to employ the plug. I have known death ensue from its use in such an emergency, a huge layer of clot being found, on inspection, between the uterus and the membranes. But, on the other hand, in a case in which delivery was resolved on, I have seen the child lost, on having been turned, from the powerful contraction of the circle of the orifice of the womb on its neck. I attributed this to the necessity of interfering early, although certainly the safe practice for the mother in every such condition is to do so. Let her be kept cool and quiet; use no means which will prevent you knowing the full extent of the bleeding; and deliver, whatever the state of the os uteri, whenever the strength of the mother begins to be obviously compromised. It will be needful, besides paying due attention to every other step of this operation, to dilate the orifice as largely as possible before you rupture the membranes. Of the second form of bleeding which has been mentioned, that where the insertion of the placenta is partial, the cases will always do well by our merely rupturing the membranes.

As to the instances of bleeding which happen after delivery, along with the irregular contraction of the womb, they commonly happen in hysterical women; and while one of the horns of the uterus is firmly clenched, its body is so relaxed that your hand passes with freedom as far upwards as the epigastrium. Such cases usually recover after the prudent extraction of whatever may have been retained; and it is a singular fact connected with them, that the bleeding which happens bears no proportion in quantity to the relaxation and flaccidity of the uterus.

With reference to the examples of bleeding after delivery, from relaxation of the neck of the uterus, which are met with along with a firmly contracted fundus uteri in the hypogastrium, they have been frequent in my practice, and are worthy of much attention. I have known practitioners, deceived by the hardness of the uterus, and the paleness of the discharge in this condition, leave their patients, as they supposed, quite well, and be summoned soon afterwards to see them suffering from extreme exhaustion, and even dying without the external loss of blood; and other women, in like circumstances, after faintness, &c., sometimes expel a large mass of clotted blood in the absence of the medical man. I am more solicitous about this kind of uterine hæmorrhage in my own practice than about any other; and besides allowing the whole transit of the child and secundines to be accomplished if possible by the mother, and then ascertaining the degree of contraction of the os uteri, I am in the habit of deferring the application of the bandage to the mother till the child has been dressed, and of retaining my hand in the meanwhile on the hypogastrium, and a cloth, accurately applied, to the pudendum. By these means the mother regains her strength, and I ascertain whether the uterus is in a doughy or contracted state, and whether stationary above the pubis, or rising at intervals above the umbilicus; the loss of every drop of blood also can be accurately measured, especially if the patient be kept on her side, and I am ready, without any hindrance from the newly-adjusted clothes of the woman, to interpose my aid when required. Now, in many cases, at least in the more delicate nervous women of large towns, even when these precautions are observed, you will be surprised by finding that, while the uterus in the hypogastrium continues firm and small, the cloths at the vulva are soaked with a rose-colored watery discharge, without any blood. This is sometimes profuse; and when it continues, the patient becomes dissatisfied, uncomfortable, and fidgety; and should she be neglected, exhaustion, accompanied by pain in the belly or back, jactitation, fainting, and death even may ensue. The explanation of all this is, that oozing from the vessels of the os uteri has occurred, and given rise to the formation of clot, which, while it gradually expands the cervix and vagina, also induces increasing discharge from the uterine vessels; and there is no safety to the woman but in the expulsion of the coagulum, either spontaneously, or by the cautious introduction of the hand into the uterus. The necessity of a cool atmosphere and a moderate allowance only of bedclothes, in the prevention and removal of this state, is also indispensable.—*Glasgow Medical Journal.*

